# Epiretinal Membrane Detection at the Ophthalmologist Level using Deep Learning of Optical Coherence Tomography

**DOI:** 10.1038/s41598-020-65405-2

**Published:** 2020-05-21

**Authors:** Ying-Chih Lo, Keng-Hung Lin, Henry Bair, Wayne Huey-Herng Sheu, Chi-Sen Chang, Ying-Cheng Shen, Che-Lun Hung

**Affiliations:** 1 Division of Nephrology, Department of Internal Medicine, Taichung Veterans General Hospital, Taichung, Taiwan; 2 Division of General Internal Medicine and Primary Care, Brigham and Women’s Hospital, Boston, Massachusetts USA; 3 Harvard Medical School, Boston, Massachusetts USA; 4 Department of Data Science and Big Data Analytics, Providence University, Taichung, Taiwan; 5 Department of Ophthalmology, Taichung Veterans General Hospital, Taichung, Taiwan; 6 Central Taiwan University of Science and Technology, Taichung, Taiwan; 7 Rong Hsing Research Center for Translational Medicine, National Chung-Hsing University, Taichung, Taiwan; 8 Stanford University School of Medicine, Stanford, California USA; 9 Division of Endocrinology and Metabolism, Department of Medicine, Taichung Veterans General Hospital, Taichung, Taiwan; 10 College of Medicine, National Yang-Ming University, Taipei, Taiwan; 11 College of Medicine, National Defense Medical Center, Taipei, Taiwan; 12 Institute of Biomedical Sciences, National Chung-Hsing University, Taichung, Taiwan; 13 Division of Gastroenterology, Department of Internal Medicine, Taichung Veterans General Hospital, Taichung, Taiwan; 14 Institute of Biomedical Informatics, National Yang-Ming University, Taipei, Taiwan; 15 Department of Computer Science and Communication Engineering, Providence University, Taichung, Taiwan; 16 Department of Computer Science and Information Engineering, Chang Gung University, Taoyuan, Taiwan; 17 AI Innovation Research Center, Chang Gung University, Taoyuan, Taiwan

**Keywords:** Diseases, Health care, Medical research, Engineering

## Abstract

**Purpose:** Previous deep learning studies on optical coherence tomography (OCT) mainly focused on diabetic retinopathy and age-related macular degeneration. We proposed a deep learning model that can identify epiretinal membrane (ERM) in OCT with ophthalmologist-level performance. **Design:** Cross-sectional study. **Participants:** A total of 3,618 central fovea cross section OCT images from 1,475 eyes of 964 patients. **Methods:** We retrospectively collected 7,652 OCT images from 1,197 patients. From these images, 2,171 were normal and 1,447 were ERM OCT. A total of 3,141 OCT images was used as training dataset and 477 images as testing dataset. DL algorithm was used to train the interpretation model. Diagnostic results by four board-certified non-retinal specialized ophthalmologists on the testing dataset were compared with those generated by the DL model. **Main Outcome Measures:** We calculated for the derived DL model the following characteristics: sensitivity, specificity, F1 score and area under curve (AUC) of the receiver operating characteristic (ROC) curve. These were calculated according to the gold standard results which were parallel diagnoses of the retinal specialist. Performance of the DL model was finally compared with that of non-retinal specialized ophthalmologists. **Results:** Regarding the diagnosis of ERM in OCT images, the trained DL model had the following characteristics in performance: sensitivity: 98.7%, specificity: 98.0%, and F1 score: 0.945. The accuracy on the training dataset was 99.7% (95% CI: 99.4 - 99.9%), and for the testing dataset, diagnostic accuracy was 98.1% (95% CI: 96.5 - 99.1%). AUC of the ROC curve was 0.999. The DL model slightly outperformed the average non-retinal specialized ophthalmologists. **Conclusions:** An ophthalmologist-level DL model was built here to accurately identify ERM in OCT images. The performance of the model was slightly better than the average non-retinal specialized ophthalmologists. The derived model may play a role to assist clinicians to promote the efficiency and safety of healthcare in the future.

## Introduction

### Epiretinal membrane

An epiretinal membrane (ERM), also known as macular pucker or cellophane maculopathy, is a pathological fibrocellular tissue that forms on the inner surface of the retina. Clinical manifestations vary from asymptomatic cellophane-like films to fibrotic contractile membranes that result in blurred vision, monocular diplopia, micropsia, metamorphopsia, decreased visual acuity, and central vision loss^1,2^. The exact pathogenic mechanisms remain determined. One hypothesis is that a separation of the vitreous membrane from the retina, or a posterior vitreous detachment, causes inflammation-mediated proliferation of retinal glial cells, fibrous astrocytes, hyalocytes, fibroblasts, myofibroblasts, and macrophages on the retinal surface^3–5^. ERMs can be either idiopathic or secondary to retinal vascular diseases, ocular inflammatory diseases, and retinal tear or detachment^6,7^.

The incidence of ERM is 1.1% per eye-year^8^, with estimated prevalence as high as 28.9% (population-dependent)^9^. ERMs occur at higher rates in the elderly population (>65 years of age). Thus, the number of people afflicted likely increases with expanding aging populations.

ERMs are diagnosed based on clinical examination historically. In comparison, the more recently developed optical coherence tomography (OCT) has greater sensitivity^10^, and becoming the mainstay for guiding ERM diagnosis and treatment^11,12^. Spectral domain OCT is a noncontact, noninvasive imaging technique based on the spectral analysis of interference patterns of back-scattered light to form two- and three-dimensional views of living retinal tissues^13,14^. Depending on the severity of the ERM, its management involves either conservative observation or surgical intervention to peel the membrane away from the retina^15,16^. If left untreated, ERM may eventually lead to blurred vision and metamorphopsia, impairing the life quality and self-care capability of patients. OCT now plays a vital role in visualizing ERMs, determining the appropriate timing and procedures for their management, as well as the prediction of postoperative outcomes^17^.

### Computer-aided diagnosis for ocular diseases

Despite the diagnostic advantage of OCT on ocular diseases, interpretation of images is a time-consuming procedure for ophthalmologists. To accelerate the diagnostic process, several studies on ocular images were made to automate the interpretation workflow using various computer vision approaches^18,19^. Even though, there is still a lot of limitation for the conventional handcrafted feature approach to hinder the widely adoption of computer-aided diagnosis in the clinical settings.

### Deep learning in medical imaging

Deep learning (DL) is an algorithm in machine learning. It utilizes statistical and computational methodology to allow the computer to perform intelligent tasks in a data-driven manner. In recent years, due to the rapid growth of data volume and computational capacity, DL approaches have made great advancements in many fields, such as computer vision, voice recognition and nature language processing. The surprising improvement over conventional approaches has positioned DL in the mainstream technique in implementing applications of the artificial intelligence.

Due to the huge success of DL in the field of computer vision, several researchers attempted to apply the technique to medical imaging. For example, Gulshan *et al*. built an automated interpretation model for images of the retinal fundus. It detects referable diabetic retinopathy (RDR) with excellent performance (area under the receiver operating curve, AUC = 0.99)^20^. Its performance is well comparable with the assessment of ophthalmologists. Ting *et al*. later developed a DL system that can identify disorders like RDR, glaucoma and age-related macular degeneration (AMD) in a multiethnic population^21^. Poplin *et al*. also established a DL model that predicts common cardiovascular risk factors and the occurrence of 5-year major adverse cardiovascular events (MACE)^22^. Their results supported the usefulness of the DL model in detecting image characteristics perceived by human observers, as well as those more subtle abnormalities human observers do not perceive.

Regarding optical coherence tomography (OCT), DL has been used to discriminate images between age-related macular degeneration and normal retina^23^. Kermany *el al*. built a DL model that detects choroidal neovascularization, diabetic macular edema, and drusen OCT images^24^. The occlusion map further allows the DL model in assisting diagnostic decisions according to manifestations of certain features recognized as deterministic abnormality by domain experts. In addition to image classification, DL was also used to solve segmentation problem for intraretinal fluid in OCT images^25^.

### Aim of the study

DL has been used for the detection of several ocular diseases (such as RDR), but only few studies focus on the ERM identification. Sonobe *et al*.^26^ confirmed DL model outperform support vector machine (SVM) in the task of ERM detection on 3D-OCT images. However, the performance on routine OCT images was not investigated. In addition, Lu *et al*.^27^ built a DL model to detect ERM, macular hole, cytoid macular edema and serous macular detachment. The accuracy was non-inferior to domain experts but the model interpretability was not elucidated. Due to ERM is a common manifestation of OCT abnormality (especially in the elderly population), it should be fully studied and regarded as a fundamental building block in developing an OCT interpretation decision support system. The present study is aimed to determine the value of DL in model detection of ERM in retinal OCT images with more comprehensive evaluation.

## Materials and Methods

This study was approved by the Institutional Review Board of Taichung Veterans General Hospital (CE18178B) with waiver of informed consent from study participants and adhered to the tenets of Declaration of Helsinki. All collected OCT images received de-identification before further processing.

### Datasets

**Figure 1:**
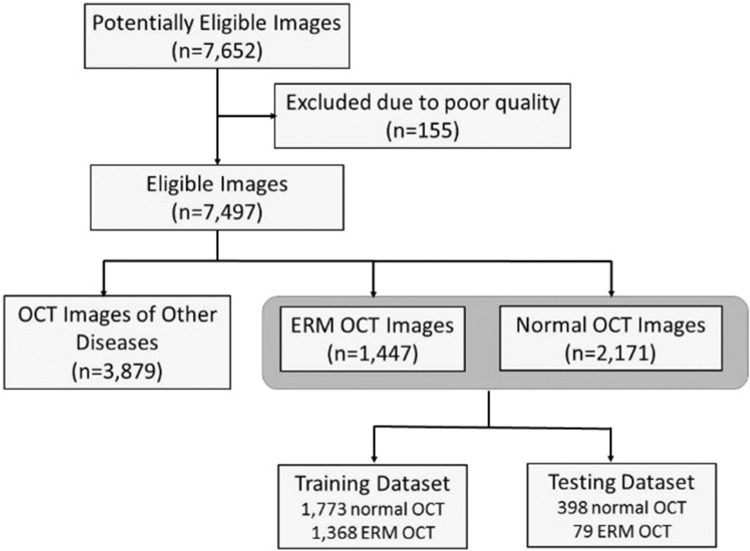
Optical coherence tomography image dataset used for the detection of epiretinal membrane. Flowchart of handling optical coherence tomography (OCT) images, showing data collection and the separation of training and testing datasets. The training dataset was used to train and validate the deep learning model.

We retrospectively collected OCT images from patients in the Taichung Veterans General Hospital between January 2010 and April, 2018. OCT studies were conducted according to recommendations of board-certified ophthalmologists based on clinical indications. The OCT images were obtained with spectral-domain OCT (Spectralis; Heidelberg Engineering, Heidelberg, Germany) and the raw image data were stored in a centralized workstation. In total, we collected 7,652 central fovea cross section OCT images from 1,197 patients. Duplicated and poor quality images were first excluded. Each OCT image was classified as normal, ERM or other ocular disease by a senior retinal specialist (with> 18 years of experience). After remove OCT images of other ocular diseases, a total of 3,618 central fovea cross section OCT images from 1,475 eyes of 964 patients were left. Normal (n = 2,171) and ERM (n = 1,447) OCT images were subsequently selected for analysis. Data were randomly split into either training dataset (n = 3,141) for training (and validation), or testing dataset (n = 477) for final evaluation of model performance to compare with ophthalmologists (see Fig. 1), and testing dataset is kept aside which is not included in the training dataset. We randomly choose 80% of the training dataset to be the actual training set and the remaining 20% to be the validation set. In order to facilitate the training process, we split the training and testing dataset in a way to let the training dataset have a more balanced class distribution (normal vs. ERM). On the other hand, we created a testing dataset with small proportion of ERM cases, that is similar to the real world OCT images data distribution. Therefore, the evaluation performance would be more likely to reflect that in the real world.

### Data preprocessing and labeling

First, the retinal specialists used a well-known open source tool, LabelImg^28^, to annotate the images as ERM or normal. In OCT images, the characteristic morphology of ERM was localized around the central fovea. All labeled images were verified by two experienced retinal specialists. The images with disagreement by the specialists were not included in the experimental dataset. Meanwhile, confusion matrix is usually used to observe the result of classification of a trained model on the training dataset after completing training process. We then performed confusion matrix to verify in case of mislabeling images to affect the classification accuracy. In this study, no images are mislabeled from confusion matrix.

### Model training, validation and testing

**Figure 2:**
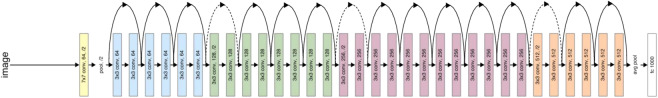
Schemtic architecture of residual network (ResNet). ResNet was composed of stacking with multiple residula block. Shortcut connections between layers were added to facilitate the training process. Currently, a number of versions of ResNet are available (such as ResNet-50, ResNet-101 and ResNet-152). In this study, we adopted ResNet-101 for modeling.

AlexNet, the state-of-the-art convolutional neural network (CNN); in designing newer network architecture is to go deeper into the data with more layers in the model. The conventional AlexNet has only 5 convolutional layers, other networks like VGG network^29^ or GoogleNet (also code-named as Inception_v1)^30^ have more layers (like 19 or 22). He *et al*. proposed a residual learning framework, called ResNet^31^, and they obtained a remarkably successful outcome in the ILSVRC 2015 competition. The key idea of ResNet (Fig. 2) is in its modeling the residual of the intermediate output, instead of the intermediate output (like in the traditional models). ResNet is able to train extremely deep networks with stochastic gradient descent (SGD) through the use of residual modules. It is also able to train a network with large amount of layers while keeping low complexity (compared with VGGNet) and it has achieved with a particular dataset a top-5 error rate of 3.57%, a performance level better than human. Currently, a number of versions of ResNet are available, with the more popular ones being ResNet-50, ResNet-101 and ResNet-152. In this study, we adopted ResNet-101 for modeling. In total, we used 3,141 OCT images for model training. Among the training datasets, 20% was used as the validation data to guide the tuning of the network hyperparameters.

The framework used to train our models is Python 3.6.4 + Keras 2.2.4 on a workstation equipped with Intel Core i7-6850K, 128 GB ram and NVIDIA GTX 1080Ti graphic card. The parameters utilized in the training were the following: learning rate, 0.0001; batch size, 32; epoch, 2000, and optimizer, Adaptive Moment Estimation (Adam).

### Statistics on testing dataset

In order to evaluate the performance of the derived model, we first calculated the area under curve (AUC) of the receiver operating characteristic (ROC) curve for the model prediction in an unseen testing dataset. Next, we determined the following as evaluation metrics for the final model: the accuracy on the training data and the accuracy, sensitivity, specificity and F1 score on the testing data. Cohen’s kappa index was used to measure the inter-rater agreement of the four ophthalmologists on the testing dataset. Confusion matrix were also generated to investigate the detail of the misinterpretation. All statistical analyses were performed using R Statistics software (v3.4.1).

### Model performance compared with clinicians

To evaluate the usefulness of the DL model in the clinical settings, four board-certified non-retinal specialized ophthalmologists of different clinical experiences were asked to interpret the unseen testing dataset which was used for the final model evaluation. Statistics with sensitivity and specificity were used to evaluate the performance of human expert on the task of OCT ERM identification. The performance of the ophthalmologists was finally compared with the DL model to validate its usefulness in the real world.

### Model visualization

To gain deeper understanding on the logic of DL model, some methods were proposed to make the prediction result more explainable. Gradient-weighted class activation mapping (Grad-CAM)^32^ is a well-known approach to produce a coarse localization map highlighting the important regions of the image that the machine learned to identify the classes. In our study, this approach was implemented before the last fully-connected layer of ResNet.

## Results

**Figure 3:**
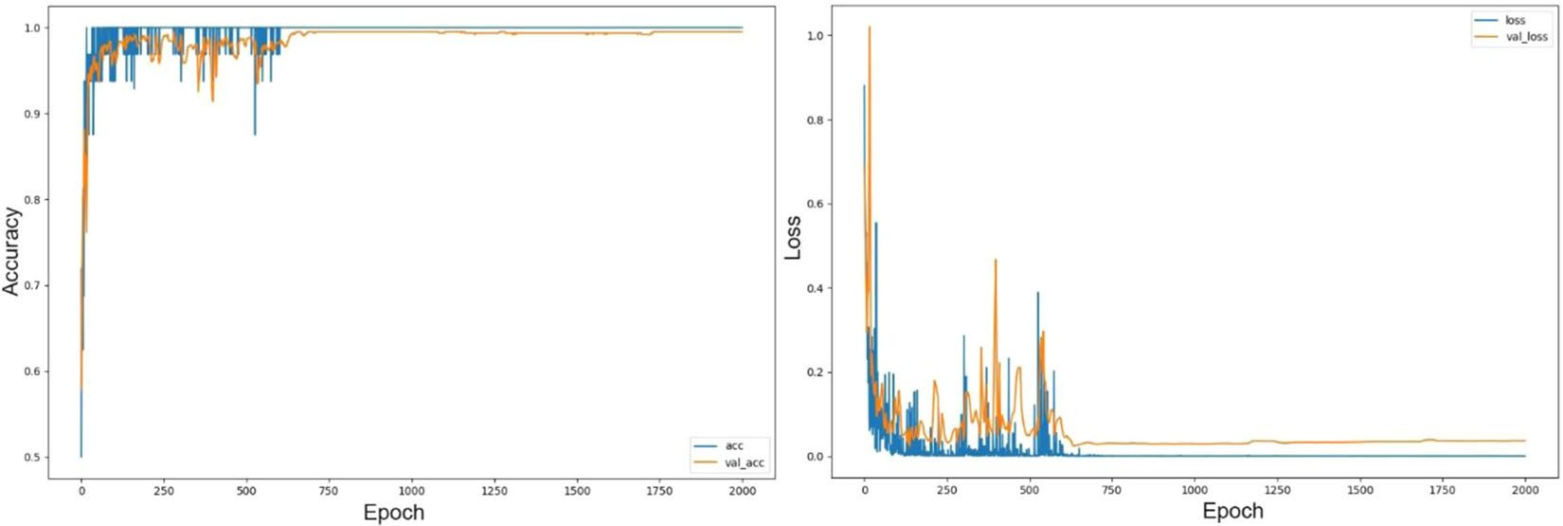
Learning curve of the derived deep learning model. The blue one is the result for the training dataset, while the orange one indicate that for the validation dataset. (Left panel: accuracy, Right panel: loss).

Finally, 3,141 OCT images were used for model training and 20% (n = 628) of them were validation dataset. During the training process, the accuracy and loss metrics were monitored and plotted as learning curves. Figure 3 shows that the model converged after 700 epochs and the training continued until 2,000 iterations. No obvious model overfitting was found. The prediction accuracy on training data was 99.7% (95% confidence interval: 99.4 - 99.9%). Due to the limitation of memory capacity of GPU device, the batch size we used is 32. Therefore, the issue of mini-batch gradient descent leaded to the spikes of loss values at the early stage before 700 epochs shown in Fig. 3.

**Figure 4:**
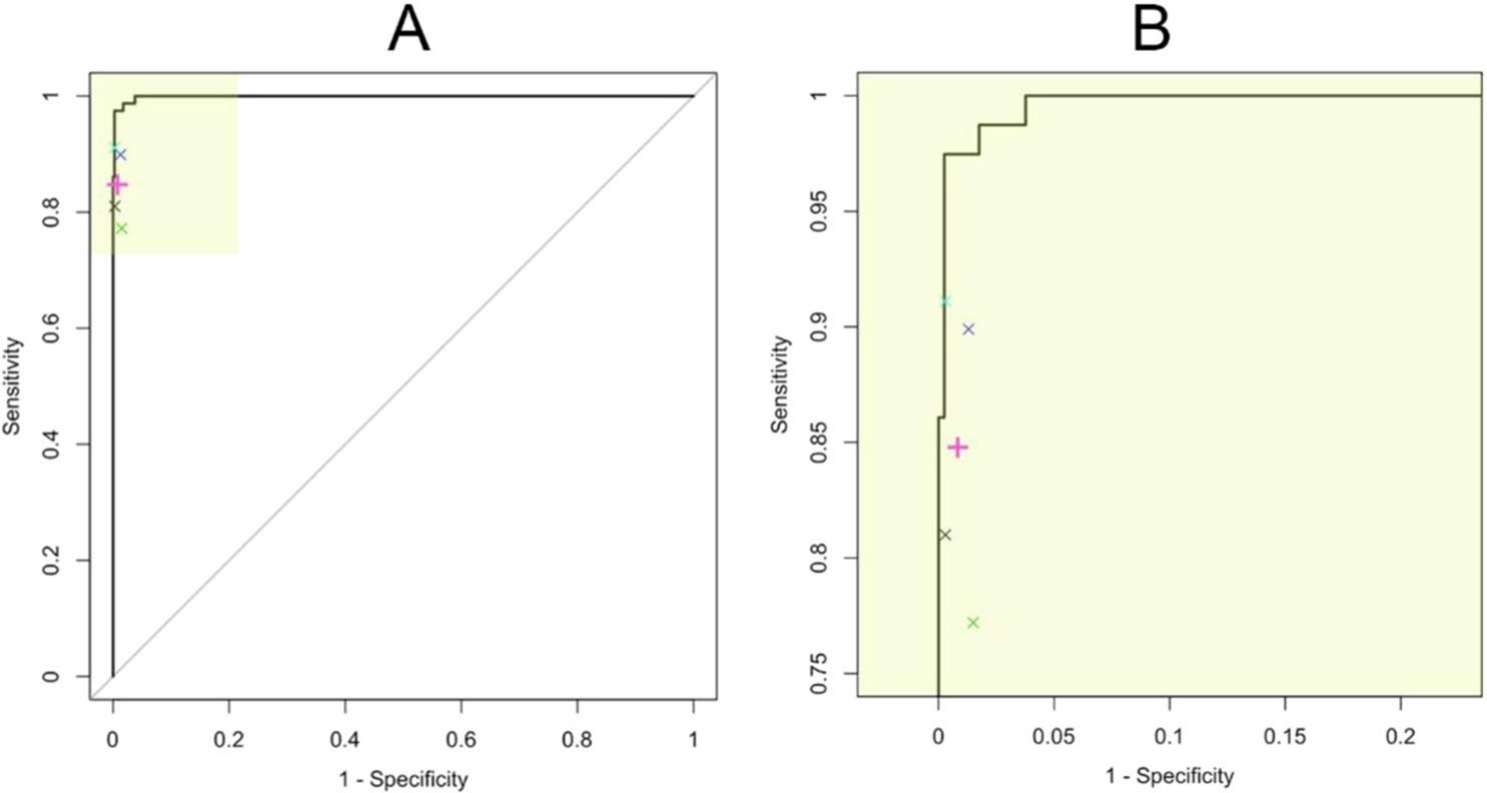
Receiver operating characteristic (ROC) curve for the identification of epiretinal membrane in the testing dataset. Evaluation results of four ophthalmologists are plotted with their average performance (pink symbol). (Panel A: original ROC curve; Panel B: close-up view of the high-lighted area in panel A).

When DL model was applied on an unseen testing dataset (n = 477), the accuracy was 98.1% (95% confidence interval: 96.5 - 99.1%). Sensitivity, specificity and F1 score on the testing data were 98.7%, 98.0% and 0.945, respectively. ROC curve of the model (AUC: 0.999) are shown in Fig. 4 together with the results of evaluation by four ophthalmologists. The close-up view (Fig. 4B) shows the DL model performed slightly better than the average of the participated ophthalmologists (pink symbol). During the error analysis, we found the DL model was more likely to result in false positive and false negative error with OCT images from myopia patients. Besides, after reviewing the false positive cases, we also identify some cases with suspicious early manifestation of ERM, indicating the derived model is quite sensitive in ERM detection. Table 1 showed the inter-rater agreement between the ophthalmologists and DL model and the confusion matrices of the clinicians’ interpretation on the testing dataset were provided in Table 2. During reviewing the disagreed images between the four ophthalmologists, we found majority of the disagreement occur in OCT images with only subtle ERM change. However, there are still few apparent misinterpretation by the clinicians noted.

**Table 1:** Inter-rater agreement* for clinicians and deep learning model.

	Clinician 1	Clinician 2	Clinician 3	Clinician 4	DL model**
Clinician 1	1.00				
Clinician 2	0.86	1.00			
Clinician 3	0.88	0.88	1.00		
Clinician 4	0.78	0.77	0.78	1.00	
DL model	0.87	0.87	0.92	0.79	1.00

*Measurement with Cohen’s kappa index.

**DL: Deep learning.

**Table 2:** Confusion matrix of the clinicians.

	Actual (+)	Actual (-)
**Clinician 1**		
Predict (+)	71	5
Predict (−)	8	393
**Clinician 2**		
Predict (+)	64	1
Predict (−)	15	397
**Clinician 3**		
Predict (+)	72	1
Predict (−)	7	397
**Clinician 4**		
Predict (+)	61	6
Predict (−)	18	392

**Figure 5:**
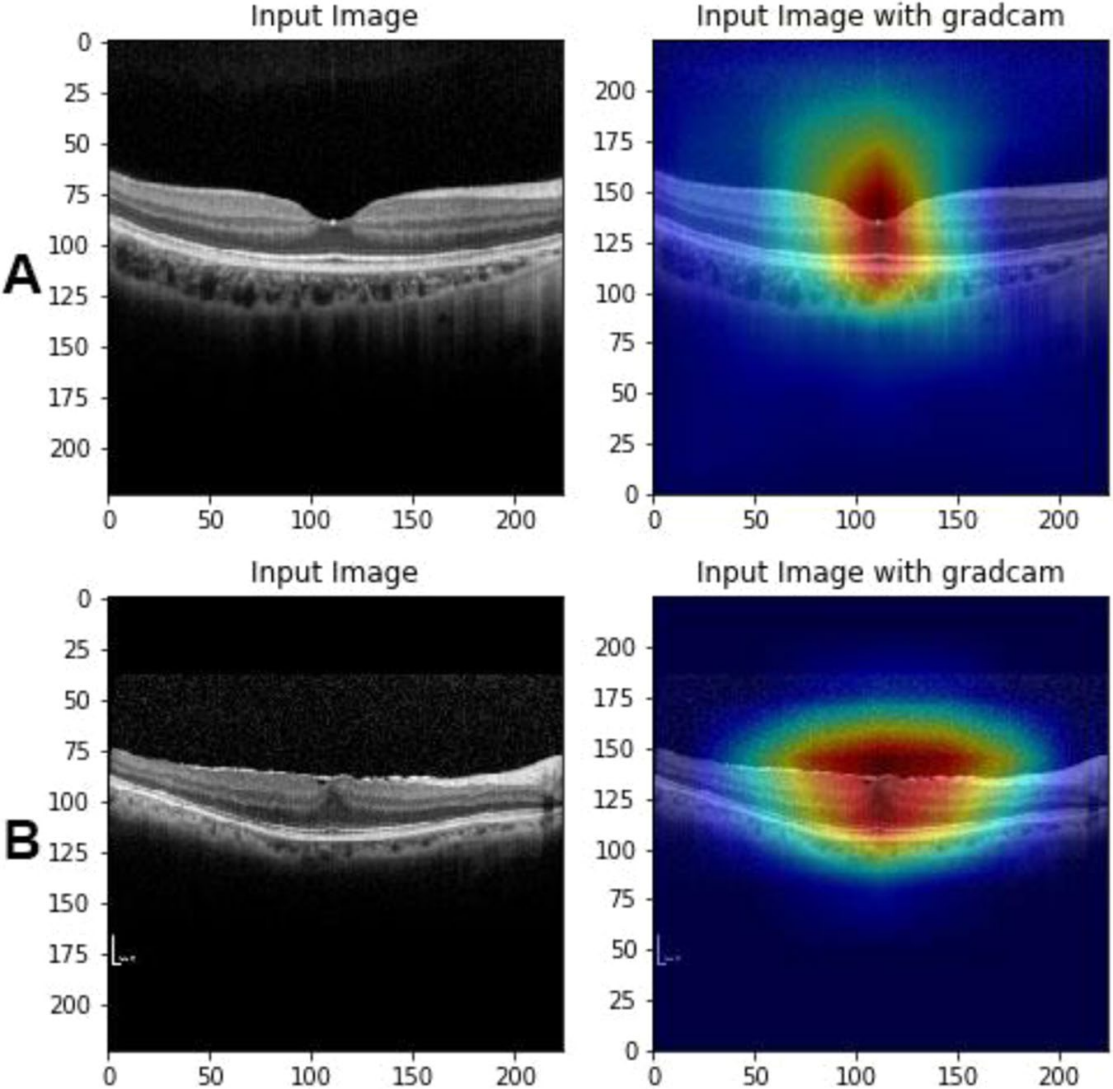
Exemplary OCT Images of normal and the epiretinal membrane (ERM) in patients. Important area for pattern recognition is highlighted with gradient-weighted class activation mapping shown on the right panels. (Panel A: normal OCT, Panel B: ERM OCT).

Figure 5A,B shows examples of normal and ERM OCT images with Grad-CAM visualization effect overlaid. Regions highlighted with warmer colors represent those areas more important for the final class determination. The ERM region of interest (ROI) was captured precisely and results are compatible with judgement of the retinal specialist.

## Discussion

Beginning with the proposal of Krizhevsky *et al*. modifications on conventional architecture of CNN were made with wider use of multiple graphics processing units (GPU) to accelerate the computational operations, DL has greatly improved results in the field of computer vision^33^. In 2016, Gulshan *et al*. of Google successfully developed a DL model that can detect RDR in retinal fundus color photography with ophthalmologist-level performance^20^. Other studies applied DL in different medical images, such as on skin, pathology, chest X-ray and electrocardiography^34–37^. Increasing evidence has indicated the potential and feasibility of utilizing DL in the interpretation of medical images.

In this study, we implemented a DL model that outperformed non-retinal specialized ophthalmologists in ERM identification. Our application can help to accelerate the process and lower the cost of ERM diagnosis. It is especially useful for regions with limited access to retinal specialist due to various reasons (such as economic issues or medical resource allocation). Further and timely referral to retinal specialist can be allocated to those whose abnormality has been detected by the DL model.

As for the diagnosis of ocular diseases, non-mydriatic fundus photography is a convenient tool of examination due to its non-requirement of pupil dilatation, and hence widely used to screen for diabetic retinopathy. Its drawback is not able to detect subtle abnormalities. Therefore, the OCT remains the gold-standard diagnostic tool for many retinal diseases. In previous studies, DL has been used to interpret and identify choroidal neovascularization (CNV), diabetic macular edema (DME), and drusen OCT images^38^. Sonobe *et al*. also confirm the superiority of DL model over SVM in ERM detection with 3D-OCT images. However, the 3D-OCT images were not supported by all the OCT imaging machine and the generalizability to routine OCT images were not investigated in their study. An OCT image DL classification model with competitive performance with domain experts were developed by Lu *et al*. However, the interpretability of the model was not elucidated. In our study, DL model showed no inferiority compared with the ophthalmologists, supporting the potential use of DL in OCT interpretation. Grad-CAM visualization confirm the validity and the robustness of the derived ERM detection DL model. ERM has not been fully studied yet but it is a prevalent disease among the elderly and is also a common finding in OCT images. An DL model for ERM identification could be an essential component in an automatic and comprehensive interpretation model for OCT. In this study, we have developed a DL model that can distinguish between ERM and normal OCT with ophthalmologist-level accuracy. We believed the established model can further improve the applicability of DL model in the highly versatile clinical settings when combined with previous developed models (like that by Kermany *et al*.) in analyzing OCT images^38^. The derived DL model may be used in the clinical settings to shorten the time period from examination to the diagnosis and increase the efficiency and efficacy of our healthcare. In addition, when the automatic DL model combine into the clinical workflow, it can also help the clinicians to avoid the occurrence of the medical error and misdiagnosis. Therefore, the derived model may also potentially play a role as a clinical decision support system to promote the patient safety in the future. In the critical period of the healthcare burden overloading, such as the COVID-19 pandemic, the DL based automatic model may also assist the clinicians to decrease the healthcare workload and prevent the healthcare providers from burnout.

## Conclusion

An ophthalmologist-level DL model has been developed here to accurately identify epiretinal membrane in OCT images. Due to the high prevalence disorders of epiretinal membrane, our model could form an essential component in automatic interpretation system for OCT images. The derived DL model may assist the clinicians to promote the efficiency and safety of healthcare in the future.
